# A multi-center inter-manufacturer study of the temporal stability of phase-contrast velocity mapping background offset errors

**DOI:** 10.1186/1532-429X-14-72

**Published:** 2012-10-20

**Authors:** Peter D Gatehouse, Marijn P Rolf, Karin Markenroth Bloch, Martin J Graves, Philip J Kilner, David N Firmin, Mark BM Hofman

**Affiliations:** 1 Royal Brompton Hospital, London, UK; 2 VU University Medical Center, ICaR-VU, Amsterdam, Netherlands; 3 University of Lund, Lund, Sweden and Philips; 4 Addenbrooke’s Hospital, Cambridge, UK

**Keywords:** Magnetic resonance imaging, Phase-contrast velocity mapping, Background velocity offset error, Cardiac output, Shunt flow, Regurgitation

## Abstract

**Background:**

Phase-contrast velocity images often contain a background or baseline offset error, which adds an unknown offset to the measured velocities. For accurate flow measurements, this offset must be shown negligible or corrected. Some correction techniques depend on replicating the clinical flow acquisition using a uniform stationary phantom, in order to measure the baseline offset at the region of interest and subtract it from the clinical study. Such techniques assume that the background offset is stable over the time of a patient scan, or even longer if the phantom scans are acquired later, or derived from pre-stored background correction images. There is no published evidence regarding temporal stability of the background offset.

**Methods:**

This study assessed the temporal stability of the background offset on 3 different manufacturers’ scanners over 8 weeks, using a retrospectively-gated phase-contrast cine acquisition with fixed parameters and at a fixed location, repeated 5 times in rapid succession each week. A significant offset was defined as 0.6 cm/s within 50 mm of isocenter, based upon an accuracy of 10% in a typical cardiac shunt measurement.

**Results:**

Over the 5 repeated cine acquisitions, temporal drift in the baseline offset was insignificant on two machines (0.3 cm/s, 0.2 cm/s), and marginally insignificant on the third machine (0.5 cm/s) due to an apparent heating effect. Over a longer timescale of 8 weeks, insignificant drift (0.4 cm/s) occurred on one, with larger drifts (0.9 cm/s, 0.6 cm/s) on the other machines.

**Conclusions:**

During a typical patient study, background drift was insignificant. Extended high gradient power scanning with work requires care to avoid drift on some machines. Over the longer term of 8 weeks, significant drift is likely, preventing accurate correction by delayed phantom corrections or derivation from pre-stored background offset data.

## Background

Phase-contrast velocity mapping is applied to blood flow [1] and myocardial velocity mapping [2] in cardiovascular magnetic resonance (CMR). Velocity images are formed by subtracting the phase images of two acquisitions with differing velocity sensitivity. As is well known, other phase differences between the two images cause stationary tissue to display an apparent non-zero velocity, known as the background offset or baseline error. This may vary gradually with position over the velocity image and underlies stationary and moving tissues. Maxwell (or concomitant) gradient effects are one cause which can be corrected analytically [3]. Eddy currents are another cause, and these are corrected to a large extent by actively-shielded gradient coils and pre-emphasis [4,5] although this is complicated by mechanical vibration effects. The remaining cause of background offset is residual errors in the pre-emphasis which accumulate phase errors during the time between velocity-encoding and echo. The background offset can sometimes be reduced by running some functions of the pulse sequence more slowly [6], but this is often incompatible with applications such as breath-hold imaging.

The background offset error may be corrected using the apparent velocity values in stationary tissue close to the vessel of interest. This correction is sufficient in most applications outside the thorax. However, for flow quantification near the heart adjacent stationary tissue is often absent. This can be solved by determining the offset in stationary tissue from the whole FOV and subtracting it using a fitted surface [7,8], thereby correcting the offset at the location of the vessel of interest. This approach is limited by several requirements: sufficient stationary tissue at adequate SNR, avoidance of phase-encode FOV wraparound, avoiding steady venous flow in the stationary tissue mask, and elimination of signal far from isocenter where the error may be highly nonlinear. All of these factors hinder reliable automatic correction by this approach.

A more basic method measures the background offset by repeating the flow study using a uniform stationary phantom after the patient, with identical sequence parameters [9], and is currently considered the “gold standard” for correction. The phantom measurements are subtracted from the patient’s velocity study to correct the background offset. This approach is simple but it is inconvenient at clinical centres and might be circumvented by a pre-stored set of corrections for a sufficiently broad range of plane orientations and image parameters. Evidently, both correction methods demand temporal stability of the velocity offset.

This work evaluated the temporal stability of phase-contrast background velocity offsets where there appear to be no previous publications.

The temporal stability of the background error might be questioned on three timescales. First, is it stable over a long period enabling the use of pre-stored corrections? Second, is the background error stable during the patient session, so that a phantom scan acquired at some time after the patient session is a reliable measurement of the error during a clinical flow study? Third, is the background error stable during all the images of a cine velocity map, even where it was retrospectively gated with continuous gradient waveform activity? The third question was confirmed long ago [10]. In the absence of published work answering the first two questions, we aimed to measure the temporal stability of background offset errors.

## Methods

### CMR test phantoms

The specification was to measure apparent velocity in a stationary uniform phantom, up to 50 mm distance from isocenter as measured in the image plane. Each centre used a different test phantom of gadolinium doped gelatine, or water which was allowed to settle for 5 minutes before beginning scanning. In one instance, to confirm the fluid’s stability, repeated phase-contrast scans were acquired during the settling time, showing that fluid settled to < 0.1 cm/s.

### CMR systems and phase contrast velocity acquisitions

The study was limited to 1.5T whole body systems as this is currently the most widely-used main field strength for cardiac CMR. Pre-emphasis for reduction of eddy current effects and automatic correction of concomitant gradient terms [3] were employed as implemented by the vendor, whereas any other filtering or correction of background offset errors was turned off because this would perform unrealistically well on large uniform phantoms. Three 1.5T scanners were used, one from each of three manufacturers (GE Signa Excite, Philips Intera and Siemens Avanto) placed in random order as scanner types 1, 2 and 3. All systems had water-cooled gradient coils and gradient amplifiers. Two of them had helium recirculation for the cryogenic magnet, while the third had no helium fills during this work (see Discussion). Each centre acquired the same single plane at 45° oblique between transverse and sagittal planes, with anterior-posterior phase-encoding, at isocentre (the aortic plane shown in reference [11]). This slice orientation is used clinically, and showed in an earlier study to be sensitive to offsets [11]. On each machine, the same plane was repeated 5 times in rapid succession during each approximately weekly session, over a period of 8 weeks. No CMR service engineering recalibrations were performed during this time. Before beginning each weekly session, a delay of at least 10 minutes without scanning was allowed. Short “scout” images were acquired each week before the first phase-contrast acquisition.

On six machines, two from each of the 3 manufacturers, a further study examined the effect of a high gradient-power sequence run continuously for at least 5 minutes between two sets of 5 cine studies as acquired above. This was acquired at a later date than the main 8-week data, following initial evaluation of that data, and two machines of each type were used to test the consistency of their behaviour.

Similar phase-contrast sequence parameters were used for each of the three scanner types, although we emphasise that the background offset values themselves were not being compared between scanners in this study, rather their drift with time. It was therefore essential that no changes in sequence parameters or slice locations were made during the 8 weeks. All cine phase-contrast acquisitions were retrospectively gated pulse sequences, where the phase-encode group was updated by each simulated ECG R-wave. The continuous gradient activity of this approach, with no silent gap while waiting for the next R-wave, has the advantage of a more stable background offset during the cardiac cycle [10]. Multiple signal averages were applied to ensure adequate SNR for measurement of the small velocity offsets. Some aspects of the pulse sequence were beyond our control using standard clinical sequences, and these are listed below for each type of scanner. Unless stated below, the velocity encoding was asymmetric (i.e. it used phase-subtraction of velocity-compensated and velocity-encoded sequence repetitions). For all of the sequences, the gradient-echo was asymmetric (i.e. the gradient-echo rephased early in the ADC sampling window for short TE). The TR values stated were between the RF excitation pulses. The parameters used for the different systems are shown in Table 1:

**Table 1:** Pulse sequence parameters

	**Scanner type 1**	**Scanner type 2**	**Scanner type 3**
Slice thickness [mm]	6	6	6
TR, TE [ms]	5.9, 3.0	5.7, 3.1	5.8, 2.3
Bandwidth [Hz/pixel]	326	355	355
Pixel size (acq) FExPE [mm]	1.25 x 2.5	1.2 x 2.4	1.25 x 2.5
FE pixels (excl oversampling)	256	288	256
PE pixels (acq)	128	138	128
Averages	2	4	2
Raw data [lines/cycle]	6	6	6
Flip angle [deg]	30	30	22

Four averages were required on scanner type 2 for sufficient SNR, possibly due to a longer phantom T1 value and/or distance from the array coils. This study did not aim to compare sequences or image SNR values.

GE Signa Excite at VUMC Amsterdam NL, and Addenbrooke’s Hospital Cambridge, UK. The readout ADC bandwidth was 41.67 kHz (pixel bandwidth 326 Hz/pixel). This used symmetric velocity-encoding (i.e. two sequence repetitions with positive and negative velocity sensitivities around the velocity-compensated waveform, also known as “balanced” velocity-encoding). The “flow analysis” flag was on, disabling a spatial high-pass filter used for phase-contrast angiography background suppression. The GE “flow optimization” control resulted in longer TE and TR than the other scanners and was therefore not used. Twenty cardiac phases were reconstructed in a 600 ms simulated R-R interval (*i.e.* temporal interpolation was applied by reconstruction).

Philips Intera. Both systems were at Lund University Hospital, Sweden. Pixel bandwidth 355 Hz/pixel (Fat/Water Shift 0.62 pixels). The “Gradient mode” was set to “Default” and “PNS mode” to “Moderate”. The slice-selective RF pulse used an asymmetric design with a late centre. The background phase-offset correction (“LPC filter”) was obviously switched off for this study. Fifteen cardiac phases were reconstructed (*i.e.* temporal interpolation was not performed during reconstruction).

Siemens Avanto. Both systems were at Royal Brompton Hospital, London, UK. The controls for RF pulse and gradient mode, which control the use of faster and stronger RF and gradient pulses, were both set to “Normal” mode. This sequence was run ungated to enable a rapid weekly test of background errors in multiple image planes at multiple slice shifts from isocenter, i.e. it did not produce a cine for each slice. The images from all other planes except the transverse-to-sagittal slice were subsequently left unused, but the total session time for the 5 repeated scans was similar to that of the other scanners. For assessment of the temporal variability during a cine sequence, a cine version of the above sequence was run once. The cine reconstruction interpolated 20 cine frames into the 1 second simulated R-R interval.

### Image analysis

**Figure 1:**
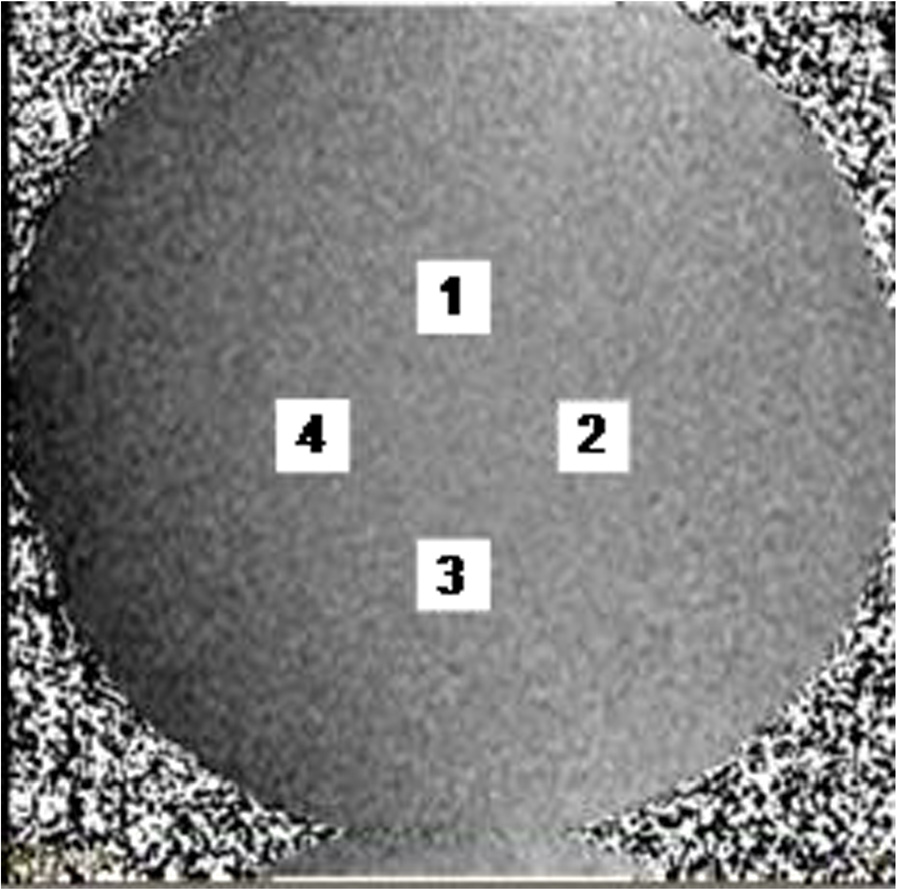
The four ROIs placed automatically on each velocity image during analysis.

All images were analyzed using software written in Matlab (The MathWorks, Natick, USA). The software reported the velocity offsets measured as the mean within square ROIs (of the same areas as circular ‘great vessel’ regions of 30 mm diameter) [12] centered 50 mm from the centre of each velocity map, which was at the isocenter. The Matlab program automatically placed and sized four such regions on each image, i.e. 50 mm to left and right, and 50 mm above and below, the centre of the image (Figure 1). The velocity offsets were measured at all 4 locations, aiming to detect temporal variations occurring in any direction from image centre (assuming those of shape *xy* over the image columns *x* and rows *y* to be negligible).

Two timescales of variations in the offsets were considered: among the 8 weekly sessions, and during each session of five scans. For these purposes, the mean over all the frames of cine acquisitions was taken for each ROI and the standard-deviation over these frames was also recorded. The standard-deviation over the cine frames was used only to check whether the background offset variations exceeded the level of random noise and systematic errors in the retro-gated cine reconstruction.

The following procedure was adopted for the purpose of determining a significant background drift. The largest peak-to-peak differences in the offsets were measured within each group of 5 scans and also across all 40 scans. This gave the largest error which could have occurred if the stationary phantom correction method had been used at any time during the weekly scans or the full 8 weeks of data collection. The errors were compared against the 0.6 cm/s offset which was suggested [11] as liable to affect some parameters derived from clinical flow measurements.

## Results

**Figure 2:**
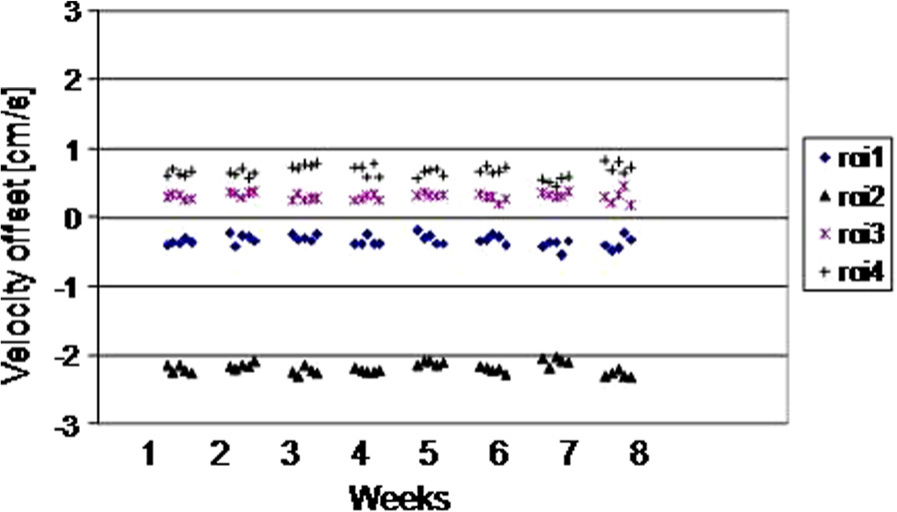
(Scanner 1) The ROI mean values on each of the 5 scans in each weekly session, for weeks 1–8.

**Figure 3:**
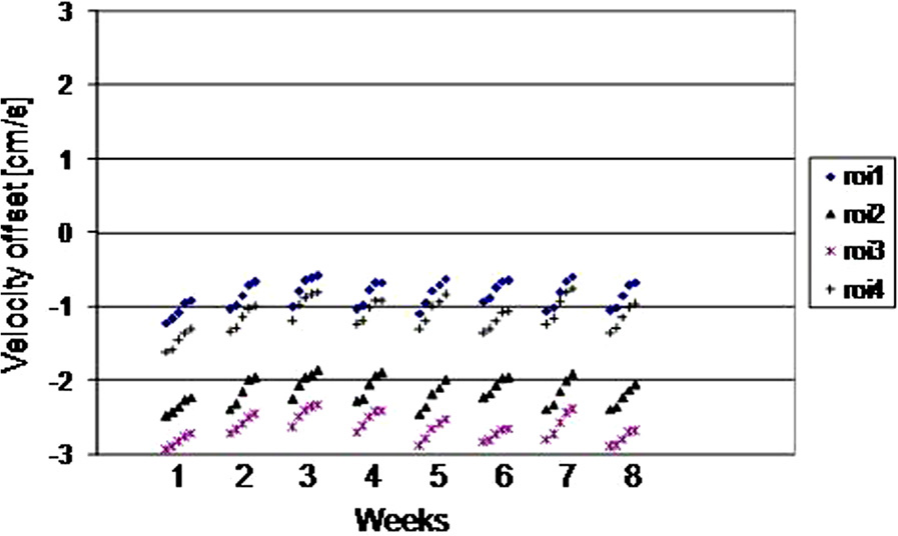
(Scanner 2) The ROI mean values on each of the 5 scans in each weekly session, for weeks 1–8.

**Figure 4:**
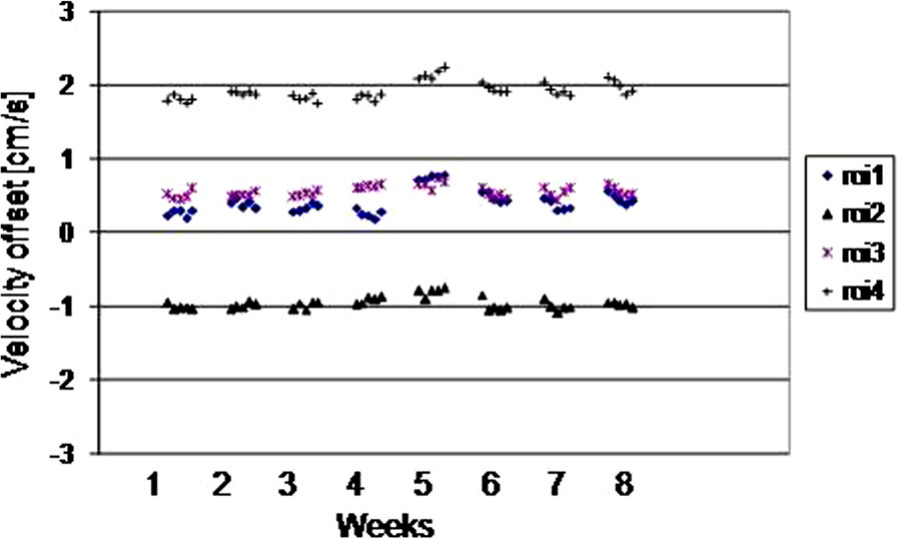
(Scanner 3) The ROI mean values on each of the 5 scans in each weekly session, for weeks 1–8.

The results for all 4 ROIs placed on the 5 scans of each of weeks 1–8 are plotted for the three scanners as Figures 2, 3 and 4. They show random variation during and between sessions, except for scanner 2 where a drift effect occurred during each session of 5 scans. Although the three graphs are all set to the same vertical axis (±3cm/s), the values themselves are not relevant in this work, rather their temporal stability.

The largest peak-to-peak differences within any 5-scan session and across all 40 scans of the 8 weeks are shown in Table 2, where the ROI value in bold is taken as the worst-case in the following text. The worst-case difference within any of the weekly sessions reached 0.28 cm/s, 0.50 cm/s and 0.23 cm/s for scanners 1 – 3 respectively, with the larger value for scanner 2 caused by the drift effect within the 5 scans of each session. Variations over the 8 week interval were larger, reaching 0.37 cm/s, 0.86 cm/s and 0.61 cm/s for scanners 1–3 respectively. The standard deviations over the 8 weeks were 0.08 cm/s, 0.21 cm/s and 0.16cm/s for scanners 1–3 respectively. The cine temporal standard-deviations were 0.13 cm/s, 0.06 cm/s, and 0.08 cm/s for scanners 1–3 respectively (largest of the 4 ROIs).

**Table 2:** Variability results of background offsets

**Scanner**	**1**	**2**	**3**
**ROI**	**1 2 3 4**	**1 2 3 4**	**1 2 3 4**
Long-term (stdev cm/s)	0.07 0.07 0.07 **0.08**	0.18 0.18 0.17 **0.21**	**0.16** 0.08 0.07 0.12
Long-term (pk-pk cm/s)	0.35 0.29 0.28 **0.37**	0.64 0.62 0.60 **0.86**	**0.61** 0.34 0.30 0.48
Session (pk-pk cm/s)	0.25 0.16 **0.28** 0.20	0.47 0.48 0.40 **0.50**	0.19 0.21 0.19 **0.23**
Cine (Sc) (stdev cm/s)	0.12 **0.13** 0.12 0.12	**0.06** 0.05 0.05 0.05	0.06 **0.08** 0.04 0.07
Spatial (Ss) (stdev cm/s)	0.94 1.0 1.1 1.2	0.20 0.18 0.32 0.21	0.23 0.32 0.22 0.28

The “Long-term” rows include all 40 scans over the 8 weeks. The “Session” row is the largest peak-to-peak variation between the 5 scans in each week. The 4 ROIs were placed at 4 locations consistently for each scan as explained in Methods. The spatial standard deviation within each single ROI is shown in the spatial row. The values in bold font are quoted in the Results as the worst-case. The symbols Sc and Ss are used in the Discussion.

**Figure 5:**
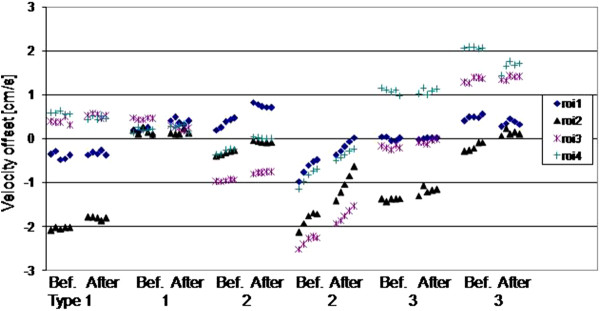
**The ROI mean values from 2 scanners of each type, before (Bef.) and after (After) high-power scanning.** The horizontal gaps for high power scanning between the groups of five scans before and after are not to temporal scale, they were approximately as long as 20 velocity cines.

The results before and after 5 minutes of high-power scanning are plotted for all 4 ROIs placed on the 5 scans, for the two systems of each of the three scanner types (Figure 5). A largely random variation occurred, except for scanner type 2 where a drift effect occurred, with some variation between machines of the same type. The largest difference in any of the ROIs after high-power scanning was 0.24 cm/s , 0.81 cm/s and 0.42 cm/s for scanner types 1,2,3 respectively (between the means of each 5-scan session).

## Discussion

Recalling the aim of reliable background offset correction for routine clinical use of phase-contrast flow measurements, we reconsider the correction methods explained in the introduction. The idea of pre-stored corrections appeared unlikely to be reliable on 2 of the 3 scanners tested, on which the peak-peak variation over 8 weeks approached or exceeded the 0.6 cm/s regarded as liable to introduce clinically significant errors in some situations. The absence of any obvious drift during the 8 weeks implies that any changes in helium levels over this time had negligible effect. Over a shorter timescale, for correction using a stationary phantom after the patient, this was reliable to well below the 0.6 cm/s limit in all scanner types, although the drift observed in scanner type 2 might eventually violate this limit.

There was no significant impact of the high power scan for scanner types 1 and 3, where the difference was <0.6cm/s. Also an earlier inter-scanner study of gradient amplifier response delays has shown high stability [13]. However, for scanner type 2, a larger change occurred. The drift effect in scanner type 2 was perhaps related to heating of system components such as the gradient amplifiers, or alterations in the residual eddy-currents, as was shown by the effect of the high power scans. This type of drift might be expected until gradient-related heating reaches a temperature steady-state against the cooling system. For the interleaved acquisition of the two sets of phase images used in the phase-contrast technique, the effects of main field-distortion known to arise from heating of the passive shim steel should almost exactly cancel. However, small sequence timing differences, even including programming errors, between the two sets of phase images may exacerbate sensitivity. Furthermore, it can be shown that gradient anisotropy (i.e. of inter-axis delays [14]) has no impact on background offset errors even for oblique planes. Probably the main factor is the temporal stability of gradient amplifier performance, and also the stability of eddy-current generation and their compensation.

The background offset error may reach clinical significance only in certain situations. The most typical of these is where the through-plane velocity data is integrated over a vessel cross-section, then summed again over all the cine frames the cardiac cycle to obtain cardiac flow measurements. The clinical impact of this error may increase with the large cross-sectional area of dilated vessels, for patients with a slow heart-rate (long R-R interval) and also for measurements derived from multiple flow acquisitions, such as the Qp/Qs ratio [9]. The maximum allowable offset of 0.6 cm/s, as considered in this study, is related to these measurements. It relates to a typical stroke volume error of less than 5%, or in terms of a Qp/Qs ratio error below 10% [11]. However, in other applications such as peak velocity assessment the background offset is usually negligible.

This study implies that correction using the clinical phase-contrast scan itself is preferable, since this would annul the question of drift during the time between clinical application and a separate correction scan, but this demands further work on the automated background correction or field-camera methods [15]. The results also suggest cautious use of phantom corrections as a reference for evaluating the performance of such methods.

For this work the relevant value for validation of robustness against noise was the variation in the mean ROI value when making repeated measurements. The spatial standard deviation (Ss) shown in Table 2 was larger and is explained as follows. The theoretical standard deviation of repeated measurements of the ROI mean (Sm) is given by the spatial standard deviation Ss divided by the square root of the number of independent pixels (Npix) in the ROI, where Npix was 226 (245 on type 2). This assumes any slope in background error over the ROI is << Ss, and also that noise is spatially and temporally uncorrelated. The value Sm gives the random noise lower limit on the standard-deviation during the cine (Sc), because Sc also includes systematic variations between frames, for example transient effects after the phase-encoding is advanced. Using the mean of Ss over the four ROIs, the values of Sm for types 1,2,3 were 0.07cm/s, 0.02cm/s, 0.02cm/s; these were substantially below the level of significance and support the robustness of this work against noise.

This study was limited to 1.5T systems as most CMR is acquired at this field strength, and there was no reason to expect a major difference in stability at other field strengths using superconducting magnets. The study was also limited to phantom acquisitions, and a more “real-life” study of changes in these after patient scanning might be more relevant. However, phantom acquisitions were essential to establish the underlying stability of the scanners. In vivo, correction algorithms are often applied. However, with the phantoms used in this study the evaluation of such algorithms is not realistic for in-vivo situations, as the most critical issue is not simulated well i.e. the lack of stationary tissue around the vessels of interest.

## Conclusion

Over the duration and activity of a typical patient study, background drift was insignificant. However, the combination of extended high gradient power scanning and work requiring background correction requires care to avoid drift on some machines. Thus, on most systems, the background offset in the velocity measurement can be corrected by a separate phantom scan within the same imaging session. Over the longer term of 8 weeks, significant drift in offset value is likely, preventing accurate correction by delayed phantom correction scans or derivation from pre-stored background offset images.

## Competing interests

Peter Gatehouse, David Firmin, Philip Kilner, Marijn Rolf and Mark Hofman work in departments with research collaborations with Siemens. Karin Markenroth Bloch is an employee of Philips. Martin Graves has a research collaboration with GE.

## Authors’ contributions

PG conceived the study. PG, MBMH, MPR, KMB enrolled and co-ordinated the multiple centers and led the design and the work. Data was acquired by PG, MPR, KMB, MJG. All authors took part in critical review and drafting of the manuscript and have read and approved the final manuscript.
